# Progress and perspectives in pediatric acute respiratory distress syndrome

**DOI:** 10.5935/0103-507X.20150035

**Published:** 2015

**Authors:** Alexandre Tellechea Rotta, Jefferson Pedro Piva, Cinara Andreolio, Werther Brunow de Carvalho, Pedro Celiny Ramos Garcia

**Affiliations:** 1 Division of Pediatric Critical Care Medicine, UH Rainbow Babies & Children’s Hospital, Case Western Reserve University School of Medicine, Cleveland, OH, USA.; 2 Department of Pediatrics, Universidade Federal do Rio Grande do Sul - Porto Alegre (RS), Brazil.; 3 Division of Pediatric Emergency and Critical Care Medicine, Hospital de Clínicas de Porto Alegre - Porto Alegre (RS), Brazil.; 4 Pediatric Intensive Care Unit, Hospital de Clínicas de Porto Alegre - Porto Alegre (RS), Brazil.; 5 Department of Pediatrics, Universidade de São Paulo - São Paulo (SP), Brazil.; 6 Division of Pediatric and Neonatal Critical Care Medicine, Hospital das Clínicas, Faculdade de Medicina, Universidade de São Paulo - São Paulo (SP), Brazil.; 7 Department of Pediatrics, Pontifícia Universidade Católica do Rio Grande do Sul - Porto Alegre (RS), Brazil.; 8 Division of Pediatrics and Pediatric Critical Care Medicine, Hospital São Lucas, Pontifícia Universidade Católica do Rio Grande do Sul - Porto Alegre (RS), Brazil.

**Keywords:** Acute respiratory distress syndrome, Definition, Respiration, artificial, Child

## Abstract

Acute respiratory distress syndrome is a disease of acute onset characterized by hypoxemia and infiltrates on chest radiographs that affects both adults and children of all ages. It is an important cause of respiratory failure in pediatric intensive care units and is associated with significant morbidity and mortality. Nevertheless, until recently, the definitions and diagnostic criteria for acute respiratory distress syndrome have focused on the adult population. In this article, we review the evolution of the definition of acute respiratory distress syndrome over nearly five decades, with a special focus on the new pediatric definition. We also discuss recommendations for the implementation of mechanical ventilation strategies in the treatment of acute respiratory distress syndrome in children and the use of adjuvant therapies.

## Abstract (pt)

A síndrome do desconforto respiratório agudo é uma patologia de início agudo, marcada por hipoxemia e infiltrados na radiografia de tórax, acometendo tanto adultos quanto crianças de todas as faixas etárias. Ela é causa importante de insuficiência respiratória em unidades de terapia intensiva pediátrica associada a significativa morbidade e mortalidade. Apesar disso, até recentemente, as definições e os critérios diagnósticos para síndrome do desconforto respiratório agudo centravam-se na população adulta. No presente artigo, revisamos a evolução da definição da síndrome do desconforto respiratório agudo ao longo de quase cinco décadas, com foco especial na nova definição pediátrica. Discutimos ainda recomendações relativas à aplicação de estratégias de ventilação mecânica no tratamento da síndrome do desconforto respiratório agudo em crianças, assim como o uso de terapias adjuvantes.

## INTRODUCTION

Acute respiratory distress syndrome (ARDS) is a widespread acute inflammatory lung injury with various degrees of intensity that occurs in response to a pulmonary or systemic insult and invariably leads to abnormalities in gas exchange (predominantly hypoxemia) and in pulmonary mechanics. It is a prototypical disease of reduced lung compliance that causes acute respiratory failure in both children and adults.^(1,2)^

The first definition of ARDS was published in 1967 when Ashbaugh et al. described a group of predominantly adult patients with various underlying diseases sharing a common progression to respiratory failure with refractory hypoxemia associated with diffuse infiltration on chest radiographs, decreased compliance and functional residual capacity, and who required the use of positive end-expiratory pressure (PEEP) to improve oxygenation. This clinical picture was attributed to pulmonary abnormalities secondary to physical and biochemical insults, with impaired surfactant function and the formation of hyaline membranes within the alveoli.^(3)^ For this reason, it was initially named “adult-type respiratory distress syndrome” due to the pathophysiological similarities with the neonatal respiratory distress syndrome (hyaline membrane disease). This initial description used vague criteria and was not specific enough to exclude other medical conditions.^(3)^

In 1988, Murray et al. suggested a more precise definition using a 4-point lung injury score including PEEP levels, the ratio of the partial pressure of arterial oxygen to the fraction of inspired oxygen (PaO_2_/FiO_2_), static lung compliance and the degree of infiltration observed on chest radiographs. Nevertheless, the lung injury score was not predictive of disease progression, and there were no specific criteria for excluding a diagnosis of cardiogenic pulmonary edema.^(4)^

More than 25 years passed before a new definition of ARDS was proposed and accepted worldwide. In 1994, the American-European Consensus Conference (AECC) defined ARDS through the following criteria: acute onset hypoxemia manifested by a PaO_2_/FiO_2_ ratio ≤ 200 in the presence of bilateral infiltrates on chest radiographs and the absence of left atrial hypertension. The concept of acute lung injury (ALI) was also introduced when, under the same conditions, the PaO_2_/FiO_2_ ratio falls between 200 and 300.^(1,5)^ The biggest criticism of this definition is that the PaO_2_/FiO_2_ ratio is considered by itself and not in relation to the level of ventilatory support employed.^(6)^ Moreover, the inclusion of radiological criteria is subject to inter-rater variability,^(7)^ and the term “acute” is not precisely defined.

Another major criticism of the 1994 consensus was the lack of definitions and concepts specific to the pediatric population. Despite knowing that there are differences between ARDS in adults and children, pediatric intensivists had to use adult ALI and ARDS criteria on their pediatric patients due to lack of a pediatric-specific definition. It is likely that this conceptual difficulty influenced the small number of studies in children with ARDS, and delayed the emergence of new concepts aimed at providing a specific definition of ARDS in children.

In 2012, a task force of the European Society of Intensive Care Medicine (ESICM), the Society of Critical Care Medicine (SCCM) and the American Thoracic Society (ATS) developed new criteria for ARDS, known as the Berlin definition; however, this definition still did not consider the pediatric population. The Berlin definition brought substantial advances such as restricting the time between the insult and the development of ARDS to seven days, improving specification of the nature of infiltrates on chest radiographs, requiring a minimum PEEP level of 5cmH_2_O to use PaO_2_/FiO_2_ ratio values in defining the severity of hypoxemia, minimizing the need for invasive measurements of pulmonary artery occlusion pressure in the absence of cardiac risk factors, and integrating ALI as a mild subgroup of ARDS based on the degree of observed hypoxemia (mild, moderate and severe) (Table 1).^(8)^

**Table 1:** The Berlin definition of acute respiratory distress syndrome

**Criteria**	**Observation**		
Timing	Within 1 week of a known clinical insult or new or worsening respiratory symptoms		
Radiological imaging	Bilateral opacities - not fully explained by lobar/lung collapse, nodules or effusions		
Origin of edema	Respiratory failure not fully explained by fluid overload or cardiac failure		
	Requires objective assessment (echocardiography) to exclude other causes of edema as etiological factors		
**Oxygenation**	**Mild**	**Moderate**	**Severe**
PaO_2_/FiO_2_	300 - 201	200 - 101	< 100
PEEP ≥ 5cmH_2_O	PEEP/CPAP/NIV	PEEP	PEEP
Estimated mortality	~ 25%	~ 35%	~ 45%

Adapted from: ARDS Definition Task Force, Ranieri VM, Rubenfeld GD, Thompson BT, Ferguson ND, Caldwell E, Fan E, et al. Acute respiratory distress syndrome: the Berlin Definition. JAMA. 2012;307(23):2526-33.^(8)^ PaO_2_/FiO_2_ - partial pressure of arterial oxygen/fraction of inspired oxygen; PEEP - positive end-expiratory pressure; CPAP - continuous positive airway pressure; NIV - non-invasive ventilation.

The Berlin definition became the new reference for ARDS in adults; however, like the AECC definition, its applicability in children remained limited since specific characteristics of the pediatric population were not considered.^(9)^ The need for invasive measurements of arterial oxygenation may lead to an underestimation of the incidence of ARDS in children, and the differences between adults and children in terms of risk factors, etiology, pathophysiology and progression were not considered in either of the two definitions.

Recently, the Brazilian Pediatric Acute Respiratory Distress Syndrome Study Group prospectively validated the use of the Berlin definition in pediatrics. The Berlin definition was superior to the AECC definition in discriminating the degree of clinical severity in children with ARDS, as evidenced by a higher mortality and fewer ventilator-free days in patients with severe ARDS compared to patients with mild and moderate ARDS.^(10)^

### Incidence

The incidence and mortality of pediatric ARDS is different than that of adults. Pediatric ARDS is relatively rare: its prevalence in children in the United States, Europe and Australia is 2-12.8 cases per 100,000 people/year.^(11-15)^ In a multicenter study involving children hospitalized in pediatric intensive care units (PICUs) in North America, 1-4% of children undergoing mechanical ventilation had ARDS.^(16,17)^ Despite the low incidence, Spanish researchers have shown that a greater number of ventilated children can develop ARDS during their stay in the ICU.^(18)^

The 1994 AECC definition has been used to identify children with ARDS, but this definition risks underestimating the actual incidence, primarily because it requires an invasive marker of oxygenation (PaO_2_). The Berlin definition does not include specific pediatric criteria but appears to have a good diagnostic performance in children younger than 24 months of age.^(19)^

Children of all ages can be affected by and develop ARDS, including full-term newborns, but its prevalence increases with age; additionally, significant differences in prevalence are rarely observed between genders. The incidence and mortality of ALI increase with age from 16 cases per 100,000/year with a 24% mortality rate at 15-19 years of age to 306 cases per 100,000/year with a 45-60% mortality rate for patients over 75 years of age.^(15)^

The high mortality rate reported in the 1980s (55 - 65%) has significantly decreased over the last 20 years (35 - 40%). This is likely a result of changes in the approach to mechanical ventilation and improvements in the intensive support of patients because no new medications have been introduced to treat ARDS. Many studies suggest that the mortality rate in children is lower compared to that in adults and ranges between 18 - 27%. However, data from Australia suggest that child mortality from ARDS could be as high as that observed in adults (35%).^(14,17,18)^

The reasons for differences in the epidemiology of ARDS between adults and children are unclear. The infrequent use of arterial blood samples and the failure to recognize ARDS in children with lower respiratory tract infections are possible reasons for the underestimation of the prevalence of ARDS in children.

### Progression

The objectives of ARDS treatment are to diagnose and treat the underlying cause, to offer supportive therapies and to provide adequate oxygenation so as to minimize secondary lung injury and extrapulmonary complications.^(2)^ Over the years, the most significant change in the treatment of ARDS has involved ventilation strategies. Mechanical ventilation is essential in treating ARDS in both adults and children. However, ventilation itself may contribute to lung inflammation and injury, barotrauma, volutrauma, atelectrauma and biotrauma, which are characteristic of ventilator-associated lung injury (VALI).

The mechanistic understanding of VALI has led to the development of lung-protective ventilation strategies, a concept highlighted by Amato et al.^(20)^ Although there is a paucity of clinical studies in children, such strategies have been unquestionably demonstrated in large collaborative studies involving adult patients with ARDS, leading to a limitation of the plateau pressure, the use of lower tidal volumes and the application of PEEP titrated to the degree of hypoxemia.^(21)^

Data from studies involving adults with ARDS demonstrated that the use of large tidal volumes (10-12mL/kg) during mechanical ventilation causes a distribution of volume to more compliant areas, leading to alveolar overdistension in these healthy areas and new alveolar injury.^(20-24)^ Due to a lack of consensus or robust clinical studies evaluating the treatment of ARDS in children, pediatric intensivists have adopted protective ventilation strategies based on the recommendations for adults. Severely injured lungs may require a lower tidal volume, whereas less diseased lungs or those in the recovery phase of disease may require a higher tidal volume.^(25)^ The benefits of using 6mL/kg in patients with low respiratory compliance and high lung injury scores have been well demonstrated.^(26,27)^ Thus, the underlying process and the severity of pulmonary disease should be considered before limiting this value for each patient.

The tidal volume adopted in children with ARDS generally is between 5 - 8mL/kg. It should be noted, however, that unlike what has been observed in studies involving adult patients, pediatric studies have not identified a specific tidal volume cutoff associated with increased or reduced ARDS-related mortality in children.^(25,28)^ Some studies suggest that using a tidal volume near 10mL/kg could actually be safe in certain children.^(25,28)^ One possible explanation for this divergent observation relative to adult populations may be due to the etiology of ARDS in children and, particularly, to its imprecise definition. Another possibility is that in retrospective studies, the patients who received higher tidal volumes were those with increased lung compliance and a better prognosis. It is, therefore, not surprising that significant variability exists in how children with ARDS are ventilated throughout the world today.^(29-31)^

### A specific consensus for acute respiratory distress syndrome in children

#### Definition

For all of the reasons cited above regarding the need for a specific definition of ARDS in children, the Pediatric Acute Lung Injury Consensus Conference (PALICC) was created. The PALICC aimed to define pediatric acute respiratory distress syndrome (pARDS), to specify its predisposing factors, etiology and pathophysiology, to make recommendations on treatment, and to identify research priorities.^(32)^ The consensus was developed by 27 experts invited for their involvement in pARDS and pediatric intensive care research in the last five years; these experts represented 21 academic institutions from eight countries. The conferences were held over a period of two years (2012/2013) with three meetings to discuss nine pARDS-related topics and to vote on recommendations. A total of 151 recommendations were analyzed using the RAND/UCLA scale (score 1 - 9). Recommendations were considered “strong” when all experts classified the recommendation with a score ≥ 7. A recommendation was considered “weak” when at least one expert classified the recommendation with a score below 7 but the average of all votes was ≥ 7. Recommendations that were considered weak were revised and resubmitted for another round of voting. Of the 151 recommendations, 132 were considered “strong” and 19 were considered “weak”.

That document^(32)^ represented a significant breakthrough with the creation of a specific definition for pediatric ARDS (pARDS), with the following aspects receiving a “strong recommendation”:

- Age group. pARDS can affect all pediatric age groups, from the neonatal period through adolescence. Evidently, perinatal causes of acute hypoxemia are excluded, including prematurity-associated lung disease, perinatal lung injury (such as meconium aspiration syndrome, pneumonia and sepsis acquired during delivery) and other congenital abnormalities (such as congenital diaphragmatic hernia or alveolar capillary dysplasia).
- Timing. Onset of hypoxemia and radiological changes should occur within 7 days after a known clinical insult.
- Myocardial dysfunction. Patients with heart disease are not excluded. Children with left ventricular dysfunction presenting with acute-onset hypoxemia and new changes on chest radiographs not explained by left ventricular failure or fluid overload and who meet all other pARDS criteria are defined as having the syndrome.
- Chest radiographs. The presence of new infiltrates consistent with parenchymal lung disease is required for the diagnosis, even if unilateral. Further studies should be performed to standardize the interpretation of radiological findings and to reduce its variability.
- Definition of hypoxemia. It is strongly suggested that the oxygenation index (OI = MAP x FiO_2_/PaO_2_, in which MAP corresponds to the mean airway pressure) be adopted over the PaO_2_/FiO_2_ ratio (recommended in the Berlin consensus for adults) to quantify the degree of hypoxemia and to determine the severity of ARDS in pediatric patients undergoing invasive mechanical ventilation. In the event that the PaO_2_ is unavailable, the oxygen saturation index (OSI = MAP x FiO_2_/SatO_2_) can be used under the same conditions proposed for the OI (Table 2).

**Table 2:** Quantification of hypoxemia using oxygenation and oxygenation saturation indices to classify the degree of severity of pediatric acute respiratory distress syndrome in patients undergoing invasive mechanical ventilation

	**Mild**	**Moderate**	**Severe**
OI	4 ≤ OI < 8	8 ≤ OI < 16	OI ≥ 16
OSI	5 ≤ OSI < 7.5	7.5 ≤ OSI < 12.3	≥ 12.3

OI - oxygenation index, based on the formula: MAP x FiO_2_/PaO_2_; OSI - oxygen saturation index based on the formula: MAP x FiO_2_/SatO_2_. When SatO_2_ was used as a criterion for the diagnosis of pARDS, oxygen therapy should be titrated to achieve SaO_2_ ≤ 97% for the OSI calculation. In patients undergoing non-invasive ventilation, there is currently no means to stratify the severity of pARDS, which is defined in these cases by an OI ≤ 300 or OSI ≤ 264.

A significant difference between the pARDS and Berlin definitions was the discontinuation of the PaO_2_/FiO_2_ ratio to grade the severity of ARDS in favor of the OI or OSI. By adding MAP into the calculation, the effect of positive pressure on oxygenation was included more objectively. This inclusion is critical because it is well known that differences in respirator management can have a decisive influence on the PaO_2_/FiO_2_ ratio and hence on the actual incidence and severity of disease classification. Evidently, these projections and theoretical benefits of employing the OI must be confirmed in pediatric studies that evaluate their sensitivity and specificity in identifying and classifying pARDS.

### Ventilatory support in pediatric acute respiratory distress syndrome

It is interesting to note that on topics related to ventilation strategies and specific management, such as the choice of tidal volume, PEEP, recruitment maneuvers and high-frequency ventilation, the recommendations were categorized as “weak” according to the criteria adopted. In other words, it can be concluded that although ventilatory support has been used in this group of patients for more than four decades, there are still conflicting aspects of the process that await adequate scientific support.

With an 88% agreement among the expert pannel, it was recommended that a tidal volume of 5 - 8mL/kg be adopted for pediatric patients with ARDS, with additional adjustments based on the pathophysiology and lung compliance. This same ambiguity or lack of consensus regarding the uniformity of the tidal volume in pARDS patients has been observed previously,^(29-31)^ and paradoxically, some studies have shown that children ventilated with tidal volumes near 10mL/kg may even have a better prognosis.^(28)^

Clearly, this “weak recommendation” regarding the optimal tidal volume for children with pARDS is not a claim that greater or supraphysiological tidal volumes should be preferentially used. The recommendation is meant to encourage, whenever possible, the use of lower tidal volumes, depending on the particular situation: patients with very low compliance would receive a tidal volume between 3 - 6mL/kg, and in less severe cases, the tidal volume would be 5 - 8mL/kg. It should be emphasized that these values should be applied early in the course of pARDS. However, it is unreasonable to attempt to maintain the same initial tidal volume target in patients with an appropriate progression of weaning from mechanical ventilation who reach larger tidal volumes through spontaneous breathing.^(33)^

The same lack of a strong consensus is also evident in choice of plateau pressure limits. As a recommendation, slightly higher plateau pressure limit values (29 to 32cmH_2_O) are acceptable under certain circumstances, such as in patients with increased thoracic elastance (low compliance).

This lack of strong agreement on ventilatory parameters can be attributed to multiple factors, such as the lack of homogeneity in the etiology of ARDS in children. It is known that one of the major causes of pARDS is viral diseases (respiratory syncytial virus, adenovirus, etc.), which can lead to mixed lung disease, resulting in decreased lung compliance (interstitial infiltrate and alveolar damage) and the increased resistance of small airways (bronchial impairment because of edema and debris in the airway lumen). As emphasized in the consensus, parameters (pressure and tidal volume) should be adjusted according to the pathophysiology and lung compliance. In situations with small airway involvement (higher time constant and increased airway resistance), it may be necessary to use strategies that are slightly different from those recommended for situations in which only lung compliance is impaired. This is perhaps the main reason for the lack of a strong agreement regarding optimal tidal volume and plateau pressure limits, and why conflicting findings related to mechanical ventilation are observed in some studies.^(25,28-31)^

The use of PEEP is a traditional ventilation strategy that is enshrined in the treatment of ARDS. Some pediatric studies have shown that the PEEP levels used in children were significantly lower than those used in adults.^(34)^ In this consensus,^(32)^ there was an 88% agreement (considered weak by the employed methodology) to use PEEP values between 10 and 15cmH_2_O to treat pARDS defined as severe. In turn, the agreement on the recommendation to use PEEP values > 15cmH_2_O for more severe cases (provided that the plateau pressure limits are maintained) was 100%. This permissiveness in the use of PEEP reflects a new trend towards a greater acceptance of these values in pediatric patients. However, at this time, there is no way to ensure that PEEP values adjusted to FiO_2_ values are applicable across the wide pediatric size ranges, such as between larger children and young infants, due to a lack of robust scientific studies. Even if PEEP adjustments relative to disease severity, FiO_2_ and patient age are yet to be determined by future research, the current consensus supports a more liberal and permissive strategy in the application of PEEP in pARDS.

Recruitment maneuvers are recommended to improve oxygenation in severe cases that are unresponsive to gradual and careful increases in PEEP. However, the best method to perform these maneuvers has not been defined. The use of sustained inflations cannot be recommended due to lack of available data in children (weak recommendation with 88% agreement). Even in adult patients with ARDS, there are only a few studies with sufficient power to demonstrate the superiority of a recruitment maneuver over other techniques in terms of more relevant outcomes, such as duration of mechanical ventilation, mortality and ICU length of stay. Hence, it is difficult to recommend one maneuver over another, or even what duration and pressure limits that should be employed. Under the current construct and based on personal experience, we understand that measures that increase the area of pulmonary gas exchange, such as recruitment through the gradual increase in PEEP and prone-position ventilation, can be adopted in patients with very severe pARDS presenting with refractory hypoxemia.

Specifically, prone positioning has not been recommended for routine use in all cases of pARDS, but should be considered as an option in severe cases (92% agreement).^(32)^ Considering all of the advantages of the prone position observed in studies of adults with ARDS, including demonstrated effects on mortality, duration of mechanical ventilation and oxygenation, coupled with few adverse side effects, particularly in children, we believe that further studies may soon demonstrate that the prone position should also be adopted in less severe cases of pARDS.^(35-37)^

High-frequency oscillatory ventilation (HFOV) is a ventilation modality with greater acceptance among neonatologists and pediatric intensivists compared to their counterparts in adult ICUs. Recent studies on the use of HFOV in adults have shown a lack of efficacy in this population.^(38,39)^ Likewise, some pediatric studies do not support the use of HFOV in children with pARDS.^(40)^ However, in the pediatric consensus,^(32)^ HFOV is recommended as an alternative in children with hypoxemic respiratory failure refractory to conventional ventilation using a plateau pressure > 28cmH_2_O (92% agreement; weak using the method adopted). Moreover, when HFOV is indicated, the concomitant optimization of lung volume through the application of recruitment maneuvers is recommended, with a 100% agreement in the consensus (strongly recommended).

If one analyzes all of these recommendations, it is notable that within the pARDS consensus, even if not unanimous, there is a strong tendency to adopt strategies aimed at the inclusion of adjunct therapies that attempt to increase the gas exchange area, such as the prone position, recruitment maneuvers and HFOV (92, 88, and 92% agreement, respectively). Although some pediatric studies already defend these strategies, it is imperative that a greater number of robust scientific studies be conducted to provide definitive support for these therapies and their precise indications.

It is worth noting the strong recommendation that an arterial oxygen saturation between 88 and 92% should be acceptable in patients with pARDS using a minimum PEEP of 10cmH_2_O. We understand that this is a conservative recommendation based on our current knowledge and the state-of-the-art. There is reasonable scientific evidence indicating that an arterial oxygen saturation level of approximately 82-88% is safe and adequate for maintaining aerobic metabolism in patients on mechanical ventilation who are properly sedated and have good perfusion (thus a low metabolic rate).^(41)^ Therefore, the consensus reinforces a tendency not to employ exaggerated ventilatory measures, such as disproportionate increases in peak inspiratory pressure (PIP), MAP, PEEP or FiO_2_ to achieve higher saturation levels.

### Non-ventilatory strategies in pediatric acute respiratory distress syndrome

The pediatric consensus discusses and makes a number of recommendations on non-ventilatory interventions that have been employed in the management of this group of patients. Because of space limitations, we chose to highlight certain topics that received a strong recommendation (100% agreement).

The use of cuffed endotracheal tubes has broad support because these reduce air leak that can influence the delivered tidal volume, PEEP levels, and lung volume. It should be noted that an air leak around the endotracheal tube may be desirable during HFOV so as to improve ventilation and increase carbon dioxide elimination, provided it does not preclude maintenance of the desired mean airway pressure.

The routine use of nitric oxide is not recommended, except in cases of right ventricular dysfunction with documented pulmonary hypertension, or in severe cases of pARDS to temporarily improve oxygenation in an attempt to avoid or postpone the institution of extracorporeal membrane oxygenation. This recommendation mirrors the current trend based on bedside experience and scientific studies demonstrating only a transient improvement in oxygenation with the use of nitric oxide without an effect on important outcomes such as mortality, duration of mechanical ventilation and PICU length of stay. Similarly, there is also a strong recommendation against administering corticosteroids in pediatric ARDS cases due to a complete lack of scientific evidence.

Other supportive measures also received a strong recommendation with 100% agreement, including goal-directed titration of sedatives with individualized plans aimed at avoiding both over- and under-sedation,^(42)^ and the use of a neuromuscular blocking agent when sedation alone does not afford adequate ventilation and oxygenation. As with sedatives, the titration of neuromuscular blocking agents should be goal-directed, and daily “sedation holiday” periods should be performed to assess the need for its continuation and the appropriate depth of sedation. Additionally, fluid intake should be tailored to maintain intravascular volume and tissue perfusion while avoiding a positive cumulative fluid balance. A hemoglobin concentration of approximately 7g/dL was recommended as the trigger for transfusion of packed red blood cells.

## CONCLUSIONS

It took us nearly five decades to establish a definition of acute respiratory distress syndrome specifically created for children, based on an international consensus and representative of the state-of-the-art in pediatric intensive care. The new definition of pediatric acute respiratory distress syndrome creates a common language for the generation of clinical studies and exchange of information among intensivists worldwide. Several centers are already attempting to validate this new definition and correlate its severity grading with disease outcomes.

The present marks an interesting period in pediatric intensive care and the future is extremely promising. The next few years should bring about accelerated progress in our understanding of pediatric acute respiratory distress syndrome, in addition to guidance regarding management of this condition where consensus still lacks.
